# Observations on Serous Effusions into the Head, Occurring in Old Persons

**Published:** 1830-07-01

**Authors:** 


### OBSERVATIONS ON THE SEROUS EFFUSION INTO THE HEAD WHICH OCCURS IN OLD PERSONS.*

The object of the writer of this article, M. Bosc, Interne des Hôpitaux, is to prove that paralytic patients are often carried off by serous effusions into the head, and to endeavour to ascertain the symptoms which distinguish such serous effusions from others. The points to be proven are, 1st. That serous apoplexy is seldom an idiopathic affection, but consequent on organic alteration of the brain or its dependencies; that it may supervene suddenly, and without precursory signs; and that it may be accompanied by symptoms which either denote or render probable its existence.

*Case* 1. *Bicêtre Hospital,* 1827. An old man of 60, weak and worn out by age and misery, had been for two years affected with hemiplegia on the left side, which appeared suddenly without any constant precursory pains in the head. One day the patient became all at once insensible, the mouth was distorted, stertor was established, and the right side was agitated by convulsive movements. He died in a few hours.

On dissection the traces of an old apoplectic extravasation were found in the right optic thalamus and corpus striatum, and the left ventricle was greatly distended by limpid serum.

*Case* 2. *Pitié Hospital,* 1828. An old man, of nearly the same age as the former, having the same habit of body, but hypertrophy of the left ventricle of the heart, in addition, was suddenly seized in 1826, with deafness, imperfect paralysis of the left arm, and temporary incomplete loss of sense. Some time after this he felt his legs gradually bend under his weight, and the upper limbs become affected with agitation, which successively increased; he suffered, in short, from paralysis agitans. The loss of motion in the left arm continued, and formication was occasionally experienced in the limbs, when suddenly one morning he fell into a state of partial insensibility accompanied with much agitation of the limbs, and died in a few hours.

On dissection there was found a small apoplectic cyst, lined by a membrane secreting a yellowish matter; white softening of the anterior part of the spinal marrow from the fourth pair of cervical nerves to the second pair of dorsal: the ramollissement occupying the two anterior thirds of the medulla, the substance of which was transformed into a whitish uniform bouillie; an abundant quantity of serum between the membranes of the brain and in the ventricles, which were much distended.

In this case the old apoplectic extravasation produced the incomplete paralysis of the arm; the ramollissement of the cord, the agitations of the limbs; and the serous effusion the apoplectic symptoms with which the drama was brought to a close. Such at least would seem to be the dicta of human experience. We have remarked in former articles in this journal, that hemiplegia is more frequently connected with partial extravasations of blood, than universal loss of sensibility and

---

* Archives Générales, Tome xxii. Février, 1830.

the state of perfect apoplexy. This observation is confirmed by the present cases, and by daily experience, and hence it is that hemiplegia is seldom fatal in the first instance. We this day witnessed the examination of an elderly female, affected with hemiplegia after a fit unaccompanied by total loss of consciousness, in whom there were the remains of as many as five apoplectic extravasations more or less considerable. The immediate cause of her death was extensive serous effusion in and on the brain, and a latent or nearly latent peripneumony. The second proposition of our author runs:—that convulsive movements of the limbs may be absent in cases of serous effusion, and death may not be its necessary consequence.

CASE 3. *Pitié Hospital,* 1823. A man, ætat. 45, had for several years been affected with incomplete paralysis consisting of feeble contractility of the muscles, and occasional deafness. He was treated with some benefit and left the hospital in which he was, but two or three days afterwards having joined a drinking party he was seized with apoplexy, abandoned by his companions in the public streets, and not brought to La Pitié till the following day. At this time he was in a state of perfect coma and insensibility, without power of moving or sense of feeling in any of his limbs, which were not in the least contracted. The face was red, but not distorted. Antiphlogistics and derivatives succeeded in arousing him so far that he was able to relate his previous history, but gangrenous erysipelas of the leg supervened, and the patient died eighteen days after the apoplectic seizure. On dissection there was found a great quantity of serum between the arachnoid and pia mater and in the ventricles of the brain. The spinal arachnoid had been in a state of chronic inflammation, for from the 5th cervical vertebra to the 6th dorsal there were streaks of false membrane of a blackish colour, a reddish serum in the cavity of the arachnoid, and softening and injection of the spinal marrow in its anterior part.

If we compare the foregoing cases, says our author, we must be struck with the following points of coincidence —1mo, the existence of a more or less ancient organic lesion; 2ndo, recent apoplectic symptoms; 3tio, alteration of the cerebral substance, with abundant serous effusion. In the two last cases the effusion was produced by disease of the medulla spinalis, was between the membranes and in the ventricles of the brain, and gave rise to general paralysis. In the first case, on the contrary, the palsy was confined to one side of the body and the effusion was into the opposite ventricle.

The third and last proposition of M. Bosc, asserts, that convulsive movements accompanying apoplectic symptoms, do not certainly indicate serous apoplexy; they only render its existence probable in so far as the individual is a paralytic.

CASE 4. *Salpêtrière Hospital,* 1829. A woman suddenly lost her consciousness, became paralytic on the right side and presented contractions and convulsions of the left arm to so violent a degree, that restraint was necessary. Bleedings, derivatives, &c. were employed but the patient died on the 5th day.

On dissection an apoplectic extravasation was found in the left optic thalamus and posterior part of the corpus striatum, from which it had penetrated the corresponding ventricle. The more fluid portions of the blood had passed through the foramen of Monro into the right ventricle in which it had accumulated. No other morbid appearances were discovered in the brain.

This case is an example of what not unfrequently occurs in apoplectic attacks, and in the artificial apoplexies produced by injuries of the head, we allude to palsy of one side and convulsive contractions of the other. The palsy is generally on the side opposed to the pressure on the brain, the contractions on the same as the latter. Several explanations of the fact have been given by authors, but none of them are very satisfactory. In the present instance M. Bosc attributes the phenomenon to the escape of the fluid parts of the blood into the ventricle opposite to that in which the extravasation had taken place. After all, we are not convinced of the accuracy of the diagnostic symptoms of serous effusion, nor do we think it possible in some instances to distinguish during life between it and venous congestion. We *are* convinced, however, that serous effusion is a very frequent mode of terminating the lives of those who have laboured under palsy, or any organic affections of the brain of a chronic character. Effusions into the head and latent peripneumony seem to us to be the general dénouement of the drama, in patients affected with irremediable, though not absolutely in themselves fatal maladies. It is for the purpose of putting this fact in a clearer light that we have thought it worth while to notice the present paper.

